# Fast multidimensional ensemble empirical mode decomposition for the analysis of big spatio-temporal datasets

**DOI:** 10.1098/rsta.2015.0197

**Published:** 2016-04-13

**Authors:** Zhaohua Wu, Jiaxin Feng, Fangli Qiao, Zhe-Min Tan

**Affiliations:** 1 Department of Earth, Ocean, and Atmospheric Science, Florida State University, Tallahassee, FL, USA; 2 Center for Ocean-Atmospheric Prediction Studies, Florida State University, Tallahassee, FL, USA; 3 The First Institute of Oceanography, SOA, Qingdao, Shangdong Province, People’s Republic of China; 4 School of Atmospheric Sciences, Nanjing University, Nanjing, Jiangsu Province, People’s Republic of China

**Keywords:** data compression, fast algorithm, multidimensional ensemble empirical mode decomposition, principal component analysis, empirical orthogonal function, adaptive and local data analysis

## Abstract

In this big data era, it is more urgent than ever to solve two major issues: (i) fast data transmission methods that can facilitate access to data from non-local sources and (ii) fast and efficient data analysis methods that can reveal the key information from the available data for particular purposes. Although approaches in different fields to address these two questions may differ significantly, the common part must involve data compression techniques and a fast algorithm. This paper introduces the recently developed adaptive and spatio-temporally local analysis method, namely the fast multidimensional ensemble empirical mode decomposition (MEEMD), for the analysis of a large spatio-temporal dataset. The original MEEMD uses ensemble empirical mode decomposition to decompose time series at each spatial grid and then pieces together the temporal–spatial evolution of climate variability and change on naturally separated timescales, which is computationally expensive. By taking advantage of the high efficiency of the expression using principal component analysis/empirical orthogonal function analysis for spatio-temporally coherent data, we design a lossy compression method for climate data to facilitate its non-local transmission. We also explain the basic principles behind the fast MEEMD through decomposing principal components instead of original grid-wise time series to speed up computation of MEEMD. Using a typical climate dataset as an example, we demonstrate that our newly designed methods can (i) compress data with a compression rate of one to two orders; and (ii) speed-up the MEEMD algorithm by one to two orders.

## 1. Introduction

Since the start of the digital era, marked by the invention of the transistor in 1947 that led the way to digital computers, the new technologies have facilitated the exponential growth of computational power and data storage capacity. While these advances have revolutionized the way to extract useful information from ever-growing data, it is still a challenge to deal with high volume, high velocity and/or high variety data and the community is still calling for (i) effective methods to store data so that the capacity limitation of local storage is not reached, (ii) fast data transmission methods that can facilitate access to data from non-local sources and (iii) fast and efficient data analysis methods that can reveal the key information from the available data for particular purposes. These demands are interwoven, as an effective storage method can facilitate fast data communication and speeded analysis and the resulting new understanding helps to determine what part of the data and how they may be effectively stored without much useful information lost.

In climate science, these demands are more evident. It is very hard in these days for a climate scientist to conduct isolated studies. The composition and maintenance of a unified and quality-controlled global domain climate dataset, such as surface air temperature, from spatio-temporally scattered observation is not an easy process. It takes a tremendous amount of expertise and continuing effort to accomplish such a job, which has led to the climate research community leaving this type of task to some specialized research centres, such as the Met Office Hadley Centre, UK, and the National Climate Data Center, USA. In recent decades, more international collaboration in understanding global climate variability and change has resulted in the formation of international data repositories to store outputs of diverse model simulations from a large group of the Earth system models developed in different countries. The typical size of model outputs for model comparison and for understanding climate variability and change in these days is of the order of petabytes. Such types of comparison and understanding projects are usually carried out by numerous groups of climate scientists located all over the world. It is not economic and feasible for a local group to store all the model outputs or even the observational data. When research using these data is carried out, a typical intermediate step is to download a fraction of data through the Internet. In this sense, fast and effective data transmission becomes important.

In this paper, we summarize our continuing efforts in addressing the two questions mentioned above, with climate data analysis used as examples. The content of the paper includes three topics: (i) the multidimensional ensemble empirical mode decomposition method (MEEMD) that aims at extracting more physically meaningful information of climate system information on different naturally selected timescales; (ii) a climate data compression method that uses principal component analysis (PCA, which is widely called empirical orthogonal function (EOF) analysis in the climate research community) to compress climate data, and (iii) a fast MEEMD algorithm that combines MEEMD and PCA/EOF-based compression. Many key ideas reported in this paper may not be new to some of the readers. However, we here make efforts to put them together in a unified framework and from an integrated perspective of both data compression and fast data analysis. In this sense, the paper should serve scientists both inside or outside the climate research community.

The paper is arranged as follows: §2 introduces MEEMD; §3 describes the basis of PCA/EOF-based data compression of climate data; §4 explains fast MEEMD. A short summary and discussion are given in the final section.

## 2. The multidimensional ensemble empirical mode decomposition

There are two major questions to be considered before the analysis of any data: (i) what information an analyst intends to obtain from data and (ii) whether a selected method is able to extract desired information from the data. A general answer to the first question is hard to get. From a physical scientist’s perspective, the desired information from any data is the quantities of the data that reflect the key information of the state of a physical system and its evolution. Traditional statistical quantities, such as mean and standard deviation of a time series over the whole data domain, while they are often pursued provide little information of variability and change and can hardly be used to infer the evolution of a physical system. Other quantities, such as cyclicity or systematic trend, may provide some information of a changing physical system.

The second question is even harder to answer. It is well known that Fourier transform expresses a time series in terms of the sum of a set of perfectly periodic sinusoidal waves of different frequencies and amplitudes. In a general sense, Fourier transform should be a good method to identify periodicity. Unfortunately, as the frequencies of the Fourier expression of a time series are determined by the selected temporal domain of the time series and the amplitudes by the projections of the time series onto individual sinusoidal waves over the selected temporal domain, the extracted periodicities are often ‘artificial’ rather than ‘natural’: one perfect sinusoidal wave of a given frequency and amplitude may be expressed in terms of a set of sinusoidal waves of which none of the frequencies and amplitudes match those of the original perfect sinusoidal wave. Widely used straight-line fitting has similar high sensitivity to the selection of data domain. Indeed, some deficiencies of these analysis methods are rooted in the nature of these methods being ‘global domain’ methods. In addition to that, the prescribed rigidness (perfect periodicity, constant amplitude or straight line) means these methods are not efficient at expressing the possible changes in frequency, amplitude and accelerated long-term changes.

From a more general perspective, as discussed in previous studies [1–3], a data analysis method having high temporal locality supplies a necessary condition to effectively isolating physical information of data. As we have learned, the subsequent evolution of a physical system cannot change the reality that has already occurred. From a dynamical system perspective, the information of the prior evolution of a physical system can only be stored in the initial condition. These two physical constraints imply that physical information of a system is temporally local. Therefore, if the information extracted from the data does reflect the physical processes operating at a given time, then the extracted information should be a temporally local quantity and the corresponding physical interpretation within specified time intervals should also not change with the addition of new data.

### (a) The empirical mode decomposition

**Figure 1.**
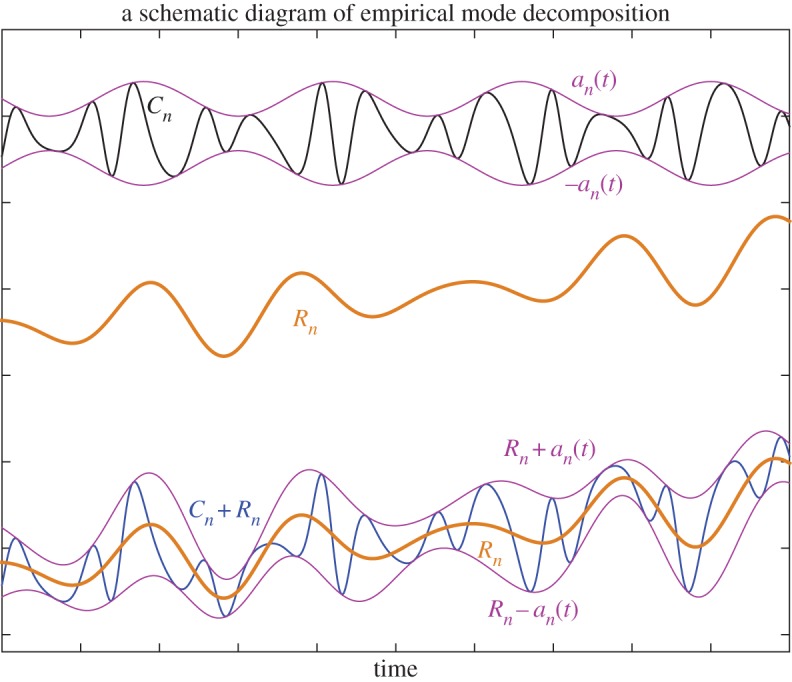
The observation behind EMD. The black line *C*_*n*_ is a pure oscillatory component (mono-component) with its amplitude *a*_*n*_(*t*) being given as the purple line at the top; the brown line is the background slower varying component *R*_*n*_ in the middle; and the blue line is the sum of *R*_*n*_ and *C*_*n*_ and the purple lines of the bottomare *R*_*n*_(*t*)+*a*_*n*_(*t*) and *R*_*n*_(*t*)−*a*_*n*_(*t*), respectively, at the bottom.

The development of the adaptive and temporally local method of empirical mode decomposition (EMD) by Huang *et al.* [4] has provided new opportunities to extract time-varying quantities from data. Before introducing the fundamentals of EMD, we first make an observation. Suppose we have starting data *R*_*n*−1_(*t*) which is composed of an amplitude-frequency-modulated oscillatory component, 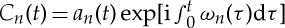, and a much slower varying function *R*_*n*_(*t*), where 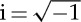; *a*_*n*_(*t*) and *ω*_*n*_(*t*) are instantaneous amplitude and instantaneous frequency, respectively, and *a*_*n*_(*t*) is non-negative and varies much slower than the wave carrier 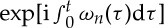 itself at any given temporal location. Such a system is schematically illustrated in figure 1. From that figure, we observe that the functions *R*_*n*_(*t*)+*a*_*n*_(*t*) and *R*_*n*_(*t*)−*a*_*n*_(*t*) serve as an upper envelope and a lower envelope of *R*_*n*−1_(*t*), respectively. Therefore, the mean of these two envelopes recovers *R*_*n*_(*t*) and the difference between *R*_*n*−1_(*t*) and *R*_*n*_(*t*) recovers the oscillatory component *C*_*n*_(*t*). It is also observed that both the upper and lower envelopes are smooth functions passing approximately through all the maxima and minima of *R*_*n*−1_(*t*), respectively.

The EMD is exactly based on such an observation and is intuitively simple. For any given data *f*(*t*), all local maxima and local minima are identified. The upper (lower) envelope passing through all the maxima (minima) is obtained using low order polynomial or spline fitting. By finding the mean of the envelopes we obtain the temporally varying reference for the highest frequency oscillatory component and then isolate it. The actual process of isolating an oscillatory component is a little more complicated and takes a refining process called ‘sifting’ as a low order spline fitting often only approximates the unknown ‘true’ envelopes (see [4–6] for more details).

By repeating this process, one obtains
2.1
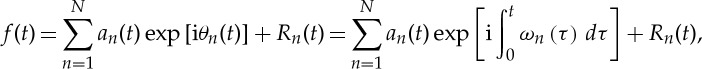
where *θ*_*n*_(*t*) is the instantaneous phase of the *n*th oscillatory component. A complete decomposition using EMD results in *R*_*n*_(*t*) being either a monotonic trend or a curved trend containing at most one interior extremum within the temporal domain of *f*(*t*) [7]. Clearly, the expression of *f*(*t*) by equation (2.1) appears to be a generalized Fourier transform that allows components to have amplitude and frequency modulation.

An important characteristic of EMD is its temporal locality, which is inherited from spline fitting through the maxima (minima) of inputted data. It can be verified using mathematical software (such as Matlab) that spline fitting has high temporal locality. As long as the number of ‘sifting’ in EMD is smaller than for example 30 and fixed, the temporal locality of EMD is highly preserved. Excessive ‘sifting’ can lead to deterioration of both locality and amplitude-frequency modulation of oscillatory components obtained using EMD [8,9]. That was also part of the reason why Wu & Huang [10] and their subsequent papers (e.g. [9,11,12] strongly recommended using a fixed number (10) of sifting.

The temporal locality of EMD naturally bypasses the non-stationarity assumption of data as analysis results are not affected by the data far away. It also automatically gets rid of excessive harmonics in expressing data because the nonlinearity in EMD is expressed by the modulations of amplitude and frequency (scale), as illustrated by simple nonlinear oscillators [4,5].

The EMD possesses some unique properties: (i) the decomposition is fully adaptive and uses no basis function and, thereby, the rigidness associated with basis function is bypassed; (ii) it is a sparse decomposition method and appears to be a dyadic filter bank [9,10,13] so the decomposition is highly effective; (3) for delta-function-like signals, EMD performs like a bank of spline wavelet of different orders [9,14]; (4) EMD components of a given noise series (except the first one) share the same Fourier spectrum [9,10,13] after rescaling frequency and amplitude; and (5) all but the first EMD components of noise have a Gaussian distribution [10]. These naturally emerged intriguing and hidden simplicities connect EMD with earlier widely used decomposition methods, such as Fourier spectrum-based filtering and wavelet decomposition. The combination of the Hilbert spectral analysis [15] and EMD is designated as the Hilbert–Huang transform by the National Aeronautics and Space Administration of the United States.

The intriguing properties of EMD and its power in extracting physical information has facilitated numerous applications in different fields of science and engineering (e.g. [16–20]), the new developments of EMD variations [21–25] and mathematical understandings of EMD (e.g. [26–28]).

### (b) The ensemble empirical mode decomposition

The locality of EMD provides a necessary condition (not sufficient) for the physical interpretability of its results. However, there are other constraints on the physical interpretability of the results from analysing non-stationary data. One of them is the sensitivity (robustness) of the analysis results to noise contained in data, as noise is ubiquitous in any real world data. If the results are not sensitive to small but not infinitesimal noise, they are generally considered physically interpretable; otherwise, they are not. As discussed in the previous section, the envelope approach in EMD designed to obtain the reference function (local mean) for the remaining highest frequency oscillatory component uses all the local maxima and local minimum; and the distances between neighbouring maxima (minima) essentially determines the local period of the remaining highest frequency oscillatory component. Therefore, the extracted oscillatory component is highly sensitive to extrema distribution. Unfortunately, unknown non-stationary noise contained in data can change the extrema values and locations of data without noise. As a complete EMD decomposition consists of continuing levels of extracting oscillatory components of lower frequency, the noise distorted results at one level can lead to continuing distortion of subsequent oscillatory components, making EMD results hardly physically interpretable. This lack of robustness essentially caused some complaints about the ineffectiveness of EMD.

To overcome this drawback, various rigid period controlling methods are designed, e.g. the intermittency test introduced in Huang *et al.* [5]. In such methods, the range of period variation in one component is prescribed and controlled. Such control often leads to excessive components, deteriorated locality and reduced adaptiveness, which calls for new and more effective approaches. The ensemble empirical mode decomposition (EEMD), a noise-assisted data analysis method, was developed exactly to meet this challenge [11].

**Figure 2.**
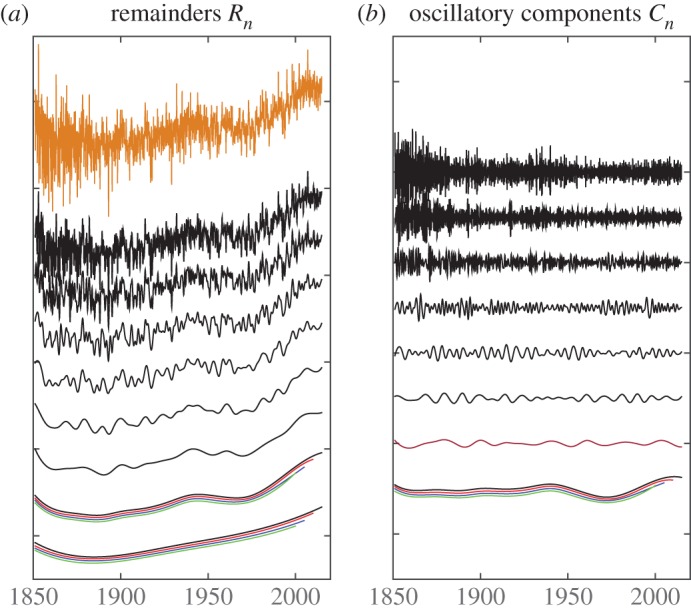
EEMD decomposition of the global annual mean land surface air temperature and EEMD temporal locality. In (*a*), the original data (brown line) and successive remainders (*R*_*j*_) after an additional EEMD component (*C*_*j*_) are extracted, i.e. *R*_*j*_=*R*_*j*−1_−*C*_*j*_ for *j*>1. In (*b*), each line represents an EEMD component (*C*_*j*_), from high frequency to low frequency. The bottom two groups of neighbouring four curves in the left panels and the bottom one group of neighbouring four curves plot the decomposition results of the same data ends in 2014 (black), 2009 (red), 2004 (blue) and 1999 (green), respectively. To illustrate clearly the results, shifts by small values of the results were carried out.

The EEMD consists of the following steps: (1) adding a white noise series to the original data *x*(*t*); (2) decomposing the data with added white noise into oscillatory components; (3) repeating step 1 and step 2 again and again, but with different white noise series added each time, and (4) obtaining the (ensemble) means of the corresponding intrinsic mode functions of the decompositions as the final result. In these steps, EEMD uses two properties of white noise: (i) temporally uniform distribution of extrema in any timescale, and (ii) EMD being effectively a dyadic filter bank for white noise [9,10,13]. The added white noise leads to relatively even distribution of extrema distribution on all timescales. The dyadic filter bank property provides a control on the periods of oscillations contained in an oscillatory component, significantly reducing the chance of scale mixing in a component. Through ensemble average, the added noise is averaged out. To some degree, the added noise mimics multiple observations of a phenomenon recorded by a single observation and serves as a ‘catalyst’ in the decomposition that leads to stable and more physically interpretable results. An example of EEMD decomposition of the global annual mean land surface air temperature (CRUTEM4) [29] is displayed in figure 2.

The decomposition displayed in figure 2 illustrates well the properties of EEMD: (i) almost no scale mixing; (ii) well-captured amplitude-frequency modulation; (iii) high adaptiveness and locality. It is also demonstrated that the decompositions of the original data and the shortened data lead to almost identical results in the overlapped temporal domain even for the very slowly varying components. In addition to that, the decomposition is dictated by the original data, which illustrates the adaptiveness, rather than by the added white noise; otherwise, the total number of components (including the trend) would be 12 instead of 9 in figure 2. From the perspective of physical interpretation of the results, the decomposition shows clearly that (i) CRUTEM4 contains a higher level of noise due to the sparsity of the observations in the 1800s [29], (ii) stronger multidecadal variability since 1930s that may be related to the strengthen of the Atlantic Meridional Overturning Circulation [15,30,31], and (iii) slowly accelerating warming trends associated with the increasing greenhouse gas concentration in the atmosphere [15]. Further study of the components of timescales of interannual or longer reveals the physical origin of each component, which will be reported elsewhere.

### (c) The multi-dimensional ensemble empirical mode decomposition

The EMD/EEMD has found wide applications in one-dimensional data analysis. However, many physical systems, including the Earth’s climate system, are spatio-temporally four dimensional. Such physical systems are often not static or periodic in spatial or temporal domains but rather evolve with different levels of spatio-temporal coherence on different timescales. An analysis method for one-dimensional data, such as EMD/EEMD, has limitation in extracting information of spatio-temporal evolution in multidimensional data. In climate science, currently there exist some matrix-based eigenvalue–eigenfunction calculation methods to deal with spatio-temporally coherent structures, such as EOF analysis [32,33], which in many other fields is called PCA, and principal oscillation pattern analysis [34,35]. In these methods, spatial structures and temporal evolutions are assumed separable and the spatial structures remain unchanged throughout the climate system evolution. However, the high sensitivity of the results obtained using these methods to both spatial and temporal domains cast shadows on the validity of this assumption. Indeed, such methods often meet difficulties in interpreting physical meanings of the results [36–38].

To bypass these difficulties, an adaptive and local method for analysing multidimensional data is desired. The development of MEEMD was to meet this challenge exactly [12]. In [12], two types of MEEMD were developed: one for spatial data (such as images) and the other for spatio-temporal data. This paper focuses on spatio-temporal MEEMD, in which a decomposition of time series at each spatial location using EEMD is first carried out and then the corresponding components from all the spatial locations are pieced together to obtain spatio-temporal evolution of components of adaptively identified timescales. This slicing approach in association with EMD was first proposed by Huang [39] and was applied in image analysis by Long [40]. However, the multidimensional EMD using the slicing approach, in general, inherits the EMD problems of scale mixing and of lack of robustness, leading to frequent sharp discontinuities between neighbouring slices.

The temporal locality and robustness of EEMD naturally overcome the discontinuity problem in the slicing approach. If the time series of two neighbouring spatial locations have spatial coherence, it is anticipated that the difference on any timescales between these two time series are small. The decompositions of these two time series using EEMD are also temporally coherent. If these time series contain a propagation signal of a given timescale, it is expected that the troughs or ridges of the corresponding components of that timescale will have a time lead or delay. Since EMD/EEMD is also a local extrema based method, such kind of signal propagation is naturally caught. Indeed, one of the advantages of MEEMD is its high capability of directly identifying the propagation signals [3,41].

**Figure 3.**
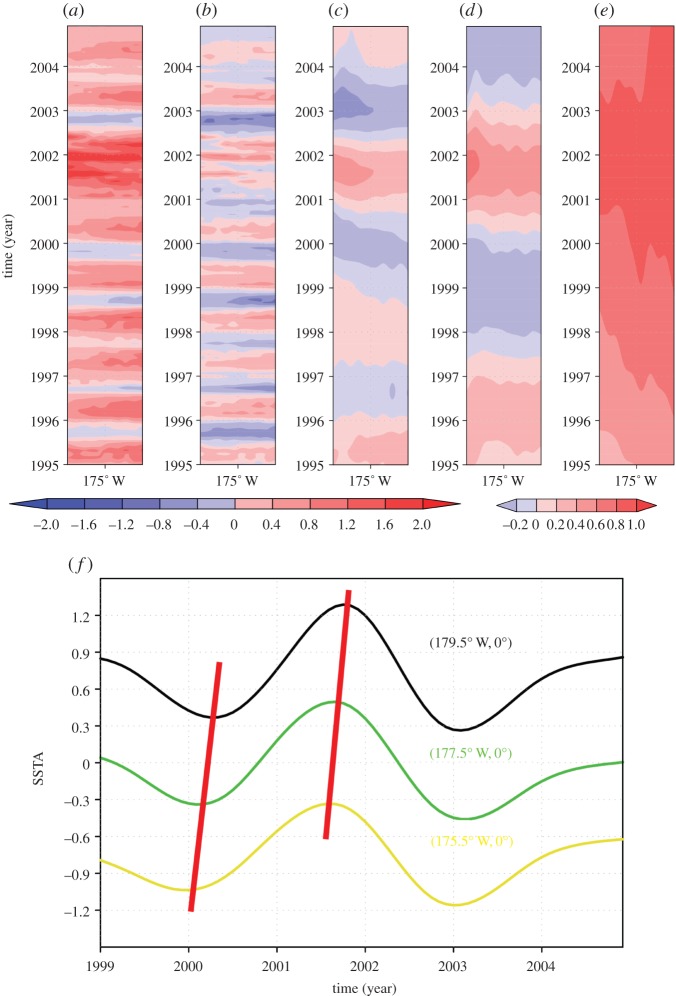
An example of MEEMD decomposition. Schematic of MEEMD analysis on SSTAs (°C; from January 1995 to December 2004) along the equator from dateline to 160° W: (*a*) original zonal–temporal evolution of SSTAs, (*b*) higher-frequency component (*C*_1_+*C*_2_+*C*_3_), (*c*) fourth component, (*d*) fifth component and (*e*) lower frequencycomponent (the sum of c6 through c10) of MEEMD. The left colour bar applies to (*a*), (*b*) and (*c*); and the right to (*d*) and (*e*). (*f*) Time series of the fourth MEEMD components (as in (*c*)) at (179.5° W, 0°) (black), (177.5° W, 0°) (green), and (175° W, 0°) (yellow). The red straight line highlights the eastward signal propagation.

Figure 3 provides an example of MEEMD decomposition of spatio-temporally gridded data. The data decomposed is the sea surface temperature (SST) from ESM2G historical run [42] provided by the Geophysical Fluid Dynamics Laboratory (GFDL) with a spatial resolution of 360 evenly distributed grid points in the zonal direction by 210 non-uniformly distributed grids points in the meridional direction (denser grid points in the tropics compared with those in the higher latitudes) and a monthly temporal resolution from January 1880 to December 2012 (a total of 1512 temporal grid points). In figure 3, only the MEEMD decomposition of SST of a randomly selected spatial domain (180°–170° W) is displayed.

The propagating signal and standing signal are well identified in *c* and *d* of figure 3, respectively. The separated standing signal and propagating signal naturally provide a preferred constant spatial pattern (often called a mode in climate science) of climate variability and a way of tracing the sources of the signal through propagation property, respectively. It is noted here that the spatially independent decomposition of data and the temporal locality inherited from EEMD make MEEMD resulted evolution of spatially coherent structure of SST variability reflect more the nature of the modelled climate system, eliminating the possibility that the spatio-temporal evolution being an artificial by-product of a mathematical method.

## 3. Climate data compression using principal component analysis

The principal component analysis/empirical orthogonal function analysis (PCA/EOF) [43] has been widely used in data analysis and image compression previously (e.g. [44,45]. In atmospheric/climate science, since Lorenz [32] introduced the PCA/EOF to atmospheric/climate research community, the applications of PCA/EOF have been largely in extracting dominant (assumed) static spatial structures of variability, such as the North Atlantic Oscillation (NAO) [46,47], Pacific–North America (PNA) pattern [48,49], Annular Mode (AO) [50,51] and El Niño/Southern Oscillation (ENSO) [52,53]; and a few applications of PCA/EOF have been designed to compress meteorological and climatological data. Recently, Feng *et al.* [54] designed a PCA/EOF compression scheme for global SST. Here, we explain further the principles behind and techniques.

Suppose, one has spatio-temporal data *T*(*s*,*t*), where *s* is spatial locations (not necessary one-dimensional originally but needed to be rearranged into a single spatial dimension) from 1 to *N* and *t* temporal locations from 1 to *M*. Using PCA/EOF, one can express *T*(*s*,*t*) into
3.1
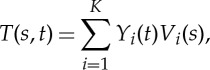
where, following the conventions in climate sciences, *Y*_*i*_(*t*) is the *i*th PC and *V*_*i*_(*s*) the *i*th EOF, and *K* is the smaller one of *M* and *N*. PCs and EOFs are often obtained by solving the eigenvalue–eigenvector problem of either temporal covariance matrix or spatial covariance matrix based on which dimensional is smaller. The variance explained by one pair of PCA/EOF is its corresponding eigenvalue divided by the sum of all eigenvalues of the covariance matrix.

The capability of PCA/EOF compression comes from the spatio-temporal coherence in data. If the data (with a size *N*×*M*) subjected to PCA/EOF is all white noise, all eigenvalues are theoretically equal and there is no preferred vector direction (the principal component) in PCA/EOF space. To retain most information of the data, one needs to retain almost all the PCs and EOFs, making the size of data in PCA/EOF expression even larger than that of the original. In the other extreme, if the original data contain only one spatial structure and oscillate with time, the original data can be expressed as the product of one PC and one EOF with a combined data size of *N* + *M*, implying that the original data of large size can be expressed by small size data without losing information, i.e. highly compressible. For climate systems, high spatio-temporal coherence results from many physical processes, such as large-scale forcing, spatial signal propagation and advection, and thermal memory; and, therefore, the data that record climate variability and change can often be expressed in terms of the sum of a small group of the products of PCs and EOFs.

To illustrate the above idea, a calculation of the efficiency of PCA/EOF in expressing the SST from the historical run of GFDL ESM2G for the whole global oceans and for different regions is summarized in table 1. In this model, the number of valid SST spatial grid points (excluding oceanic grid locations that have seasonal sea ice) is about 52 920 (*N* in equation (3.1)) and the total number of temporal locations is 1512 (*M* in equation (3.12)), resulting in a total number of values of 52 920 ×1512 (*N*×*M*). If PCA/EOF is applied to compress these data to reach an explained variance level of 98% of that of the original, 123 (*K* in equation (3.1)) PC/EOF pairs need to be retained. Each EOF has the same number of data points as the original valid spatial grids, so the total number of values of the compressed data is 123×(52 920+1512) [*K*×(*N*+*M*)]. The compression rate (which is the ratio of original data size and compressed data size) is about 12 for retaining 98% of variance. If the variance retaining rate increases to 99%, the compression rate becomes about 6.

**Table 1.** Total number of PC/EOF needed to express SST of ESM2G historical run with various prescribed variance thresholds for the whole globe and for different regions. Global, represents the whole global domain; NPac, north Pacific; TPac, tropical Pacific; SPac, south Pacific; NAtl, north Atlantic; TAtl, tropical Pacific; SAtl, south Atlantic; TInd, tropical Indian Ocean; Sind, south Indian Ocean. The division of these regions does not follow exactly the geographical convention, which is displayed in figure 4. The tropical Pacific is further divided into western tropical Pacific (WTPac) and eastern tropical Pacific (ETPac).

	no. eigenfunctions required										
variance explained	Global	NPac	ETPac	WTPac	SPac	NAtl	TAtl	SAtl	TInd	SInd	TPac
98%	123	16	36	28	19	13	19	16	46	22	52
99%	257	38	83	65	41	20	40	35	101	49	123

The above calculation does not take into account the nature of climate variability. Although climate data contain high spatio-temporal coherence, it is often more regional than global: for example, the SST variability over the whole north Atlantic follows, to a significant degree, a tripole spatial structure [55] on interannual timescale, while the tropical Pacific SST on the interannual timescale has the dominant shape of a tongue extended from the Pacific east coast to about the dateline. However, the temporal variability of the north Atlantic SST has little correlation with the temporal variability of El Niño in the tropical Pacific. Such global scale incoherence leads to PC/EOF components accounting for the dominant variabilities of different regions being located in different subspaces of PC/EOF space. When the global domain is selected for PCA/EOF, it is expected that the accumulated variance increment associated with increasing number of PC/EOF components is slow, making the PCA/EOF-based compression less efficient.

**Figure 4.**
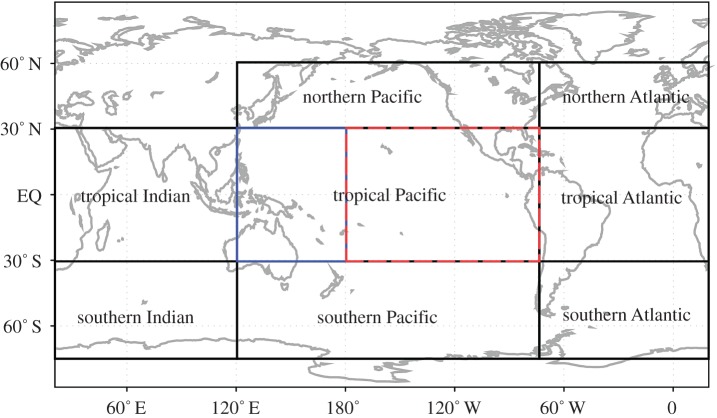
Division of global oceans. Black open boxes denote the main nine basins: northern Atlantic Ocean (75° W–20° E, 30° N–60° N), northern Pacific Ocean (120° E–75° W, 30° N–60° N), southern Indian Ocean (20° E–120° E, 75° S–30° S), southern Atlantic Ocean (75° W–20° E, 75° S–30° S), southern Pacific Ocean (120° E–75° W, 75° S–30° S), tropical Indian Ocean (20° E–120° E, 30° S–30° N), tropical Atlantic Ocean (75° W–20° E, 30° S–30° N) and tropical Pacific Ocean (120° E–75° W, 30° S–30° N). The tropical Pacific Ocean is further divided into tropical eastern Pacific (180°–75° W, 30° S–30° N, red dashed box) and tropical western Pacific(120° E–180°, 30° S–30° N, blue dashed box).

One way to improve the efficiency of the representation of data in terms of PC/EOF component is to divide the global spatial domain into a set of sub-regions. Such type of division of global domain into sub-regions was adopted in earlier studies [56,57]. The physical rational behind this mathematical approach is that the climate variability of a smaller region tends to be more spatio-temporally coherent than a bigger region containing that smaller region, and, therefore, it is expected that fewer PC/EOF components are required to account for a threshold level of variance of the climate variability of that region. If we divide the original global spatial domain into *n* sub-regions containing *N*_1_, *N*_2_,…,*N*_*n*_ spatial grids, respectively, with all *N*_*i*_, *i*=1,…,*n*, greater than *M*, we anticipate that the numbers of the retained PC/EOF pairs for all sub-regions *K*_1_, *K*_2_,…,*K*_*n*_ are all smaller than *K* for the same prescribed accumulated variance level. As we mentioned earlier, the total number of data values in PCA/EOF representation of the original data of the global spatial domain by equation (3.1) is *K*×(*N*+*M*). For the new approach of using spatial division, the total number of values in PCA/EOF representation is
3.2
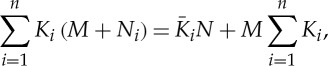
where

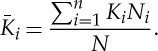
Therefore, the actual compression rate CR is
3.3
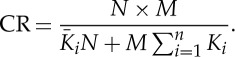
In figure 4, a non-equal area division of global oceans is made. This division follows largely the understanding of climate dynamics occurring in these regions. As shown in table 1, the number of PC/EOF pairs needed to account for 98% of variance is less than 40 for almost all sub-regions. If we select 40 PC/EOF pairs for all regions to compress SST, the compression rate is about 30 for the whole globe. More details of the compression rates for sub-regions are shown in the top panel of figure 6. It is noted that the variability associated with the excluded PC/EOF pairs behaves like white noise in time and has fine structures in space. It is also noted that an optimized division and an optimized selection of PC/EOF pairs for each region would lead to a higher rate of compression.

## 4. The fast multidimensional ensemble empirical mode decomposition

As is known, for a time series of length *M*, the algorithm complexity of cubic spline fitting through its local extrema is about *O*(*M*), and so is that of the EEMD as it only repeats the spline fitting operation with a number that is not dependent on *M*. However, as the sifting number (often selected as 10) [10,11] and the ensemble number (often a few hundred) [10,11] multiply to the spline fitting operations, the EEMD is time consuming compared with many other time series analysis methods. In MEEMD introduced in the previous section, as it employs EEMD decomposition of the time series at each grid, the EEMD operation is repeated by the number of total grid points of the domain. For the model data we compressed in the previous section, we need to repeat 52 920 times of EEMD calculations, making the computational burden worse. As introduced earlier, the spatio-temporal consistency of MEEMD is inferred from the temporal locality and robustness to noise of EEMD and the assumption that the difference between the time series of neighbouring locations are small on all timescales. While the logic behind is solid, an alternative spatio-temporal coherent decomposition would still be helpful to potential MEEMD users of different backgrounds. In answering these challenges, a fast MEEMD was developed [54].

The idea of the fast MEEMD is very simple. As PCA/EOF-based compression expressed the original data in terms of pairs of PCs and EOFs, through decomposing PCs, instead of time series of each grid, and using the corresponding spatial structure depicted by the corresponding EOFs, the computational burden can be significantly reduced.

**Figure 5.**
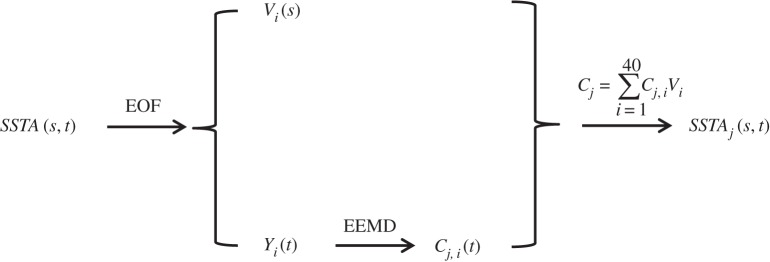
Flowchart for the fast MEEMD. *V*_*i*_(*s*) and *Y*_*i*_(*t*) denote the *i*th EOF and the *i*th PC after the *SSTA*(*s*,*t*) is subjected to EOF analysis of SSTA, respectively. The subscripts *j* denotes the *j*th EEMD component of *Y*_*i*_(*t*) from EEMD or *j*th MEEMD component after the rearrangement (corresponding to the rightest arrow) is carried out.

The fast MEEMD is schematically illustrated in figure 5, which includes the following steps: (i) all pairs of EOFs, *V*_*i*_, and their corresponding PCs, *Y*_*i*_, of SSTA over a compressed sub-domain are computed; (ii) the number of pairs of PC/EOF that are retained in the compressed data is determined by the calculation of the accumulated total variance of leading EOF/PC pairs (e.g. 99%); (iii) each PC *Y*_*i*_ is decomposed using EEMD, i.e.
4.1
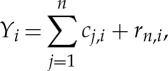
where *c*_*j*,*i*_ represents simple oscillatory modes of certain frequencies and *r*_*n*,*i*_ is the residual of the data *Y*_*i*_. (iv) The final result of the *i*th MEEMD component *C*_*j*_ is obtained as
4.2
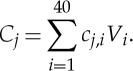
In this compressed computation, we have used the approximate dyadic filter bank properties of EMD/EEMD. It is expected that the same rank EEMD components of different PCs of the compressed data usually stay within similar filter windows.

Table 1 indicates that only 257 PC/EOF pairs are needed to retain 99% of variance of the original data of whole global oceans. If the computation burden of PCA/EOF analysis and of projection back onto the spatio-temporal domain is negligible compared with EEMD decomposition (which is true for this set of data), then the number of time series needed to be decomposed in the fast MEEMD for this case is only 257, less than 1/200 of the original 52 920 time series, implying potential speed-up of computation by more than 200 times.

**Figure 6.**
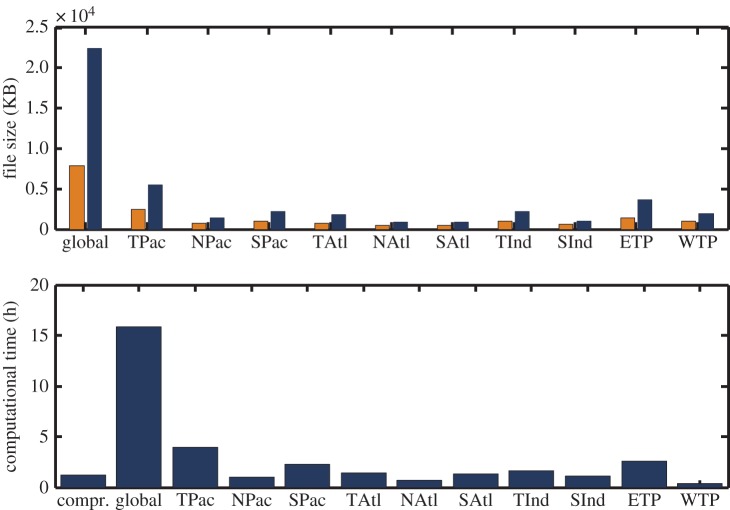
Compression and computational speeding-up rates. (*a*) File size of the original uncompressed (blue bars) and compressed (yellow bars) SSTA data for each basin; (*b*) computation time of compressed (the 1st bar) and uncompressed SSTA data in each basin (the 2nd to 7th bar). In the top panel, the blue bars are scaled to 1/10 of its actual value. Note that for the compressed SSTA data, the total computation time is independent of domain size. Similarly, the 2nd to 12thbars in lower panel (computational time with original MEEMD) are scaled to 1/10 of its actual value. On the other hand, the computational time for compressed data (approx. 1 h, 1st bar in lower panel) does not vary with different domains and it was not scaled.

An actual decomposition using fast MEEMD is carried out by applying it to the EMS2G model SST. In this calculation, we adopted the sub-region division approach illustrated in figure 4 and retained 40 PCs for each sub-region. In this case, the whole global oceans are divided into nine regions and a total of 360 PCs are decomposed using EEMD. The decomposition is carried out using a Matlab EEMD program in a Unix server. When EEMD is applied grid-wise, the total computational time for the global SST is 1600 h. However, when 40 PCs for each region are decomposed using the same program, the total computation time (including PCA/EOF calculation) is about 13 h. Therefore, a speed-up of 123 times is achieved. The speed-up rate is slightly small than 147, which is the ratio of the total number (52 920) of time series of the original data and the total number (360) of PCs retained for all nine regions. The computational speed-up rates for all sub-regions are displayed in the bottom panel of figure 6.

The remaining question is whether the MEEMD results using the grid-wise decomposition and PC-based decomposition are equivalent. To answer this question, we follow Feng *et al.* [54] to compare corresponding final components obtained using two different approaches for randomly selected grid points. It is confirmed, just as in Feng *et al.* [54], that the differences between all the corresponding components are indeed small and negligible. Owing to the limit of the length of the paper, the figure displaying these results (similar to fig. 8 in Feng *et al.* [54]) is not shown.

## 5. Summary and discussions

In this big era, both computational power and capacity of data storage are growing exponentially. Various data repository models in cloud computation require higher data transmission capacity and transmitting compressed data through the Internet. At the same time, effective analysis methods for extracting useful information from big data need to be developed. Under these circumstances, we have designed both a data compression technique and a fast algorithm for extracting evolution information from spatio-temporally multidimensional data of evolving physical systems, such as the Earth’s climate system.

The devised lossy compression method used the nature of high spatio-temporal coherence for most of the physical systems. By using the efficiency of PCA/EOF in representing spatio-temporally coherent multidimensional data, the storage size is reduced by more than an order. All the key information of the variability and change in the original data is retained in the compressed data. At the same time, this compression leaves out the spatio-temporally incoherent noise.

The fast algorithm discussed in this study is related to the most recently developed adaptive and temporally local analysis method, the MEEMD. The adaptiveness and the locality of the method enable the method to extract key evolution information of a physical system hidden in data. The newly devised fast MEEMD can loyally recover the results of the original MEEMD, but with a computational speed that is two orders faster than the original MEEMD when they are applied to decompose a typical climate dataset, such as the outputs from current Earth system model. It is anticipated that these new techniques will improve significantly the power of understanding climate variability and change through analysing climate data.
